# Defluorination of Sodium Fluoroacetate by Bacteria from Soil and Plants in Brazil

**DOI:** 10.1100/2012/149893

**Published:** 2012-04-24

**Authors:** Expedito K. A. Camboim, Michelle Z. Tadra-Sfeir, Emanuel M. de Souza, Fabio de O. Pedrosa, Paulo P. Andrade, Chris S. McSweeney, Franklin Riet-Correa, Marcia A. Melo

**Affiliations:** ^1^Unidade Acadêmica de Medicina Veterinária, Universidade Federal de Campina Grande, Avenida Universitária, s/n, Bairro Sta. Cecília, Patos, PB, CEP: 58700-970, Brazil; ^2^Laboratório de Fixação Biológica de Nitrogênio, Departamento de Bioquímica e Biologia Molecular, Universidade Federal do Paraná, Curitiba, PR, CEP: 81531-980, Brazil; ^3^Departamento de Genética, Universidade Federal de Pernambuco, Recife, PE, CEP: 50670-901, Brazil; ^4^CSIRO Livestock Industries, Queensland Bioscience Precinct, Carmody Road, 306, St Lucia, 4067, QLD, Australia

## Abstract

The aim of this work was to isolate and identify bacteria able to degrade sodium fluoroacetate from soil and plant samples collected in areas where the fluoroacetate-containing plants *Mascagnia rigida* and *Palicourea aenofusca* are found. The samples were cultivated in mineral medium added with 20 mmol L^−1^ sodium fluoroacetate. Seven isolates were identified by 16S rRNA gene sequencing as *Paenibacillus* sp. (ECPB01), *Burkholderia* sp. (ECPB02), *Cupriavidus* sp. (ECPB03), *Staphylococcus* sp. (ECPB04), *Ancylobacter* sp. (ECPB05), *Ralstonia* sp. (ECPB06), and *Stenotrophomonas* sp. (ECPB07). All seven isolates degraded sodium-fluoroacetate-containing in the medium, reaching defluorination rate of fluoride ion of 20 mmol L^−1^. Six of them are reported for the first time as able to degrade sodium fluoroacetate (SF). In the future, some of these microorganisms can be used to establish in the rumen an engineered bacterial population able to degrade sodium fluoroacetate and protect ruminants from the poisoning by this compound.

## 1. Introduction

In Brazil, thirteen species of plants causing sudden death associated with physical effort are responsible for nearly 500.000 cattle deaths each year: *Palicourea marcgravii*, *P. aeneofusca*, *P. juruana*, *P. grandiflora*, *Pseudocalymma elegans*, *Arrabidaea bilabiata*, *A. japurensis*, *Mascagnia rigida*, *M. elegans*, *M. pubiflora*, *M. aff. rigida*, *M. exotropia*, and *M. sepium *[1, 2]. Sodium fluoroacetate was identified as the active principle of *P. maracgravii *[3] and *A. bilabiata *[4] and is probably present in other plants of these genera. It disrupts the tricarboxylic acid (TCA) cycle, being first converted to fluorocitrate which in turn inhibits the enzymes aconitase and succinate dehydrogenase resulting in citrate accumulation in tissues and plasma and ultimately causing energy deprivation and death of the animal [5].

The use of sodium fluoroacetate is prohibited in Brazil, occurring exclusively as a natural product in plants. In Australia the compound is used in impregnated baits for the control of rabbit, fox, dingo, and other mammal populations; however, when introduced in the environment it can select fluoroacetate-degrading microorganisms [6].

Microbial degradation of sodium fluoroacetate is catalyzed by a haloacetate halidohydrolase, which is able to cleave the strong carbon-fluorine bond [7]. Twenty-four fluoroacetate-degrading microorganisms were isolated from soil in Central Australia, from seven bacterial genera (*Acinetobacter, Arthrobacter, Aureobacterium, Bacillus, Pseudomonas, Weeksella, *and* Streptomyces*) and four genera of fungi (*Aspergillus*, *Fusarium*, *Cryptococcus,* and *Penicillium*) [8].

The possibility to prevent fluoroacetate poisoning in ruminants by the ruminal inoculation of genetically modified bacteria, containing a gene encoding fluoroacetate dehalogenase has been investigated [9, 10]. A thorough search among samples taken from environment, such as soil, leaf, and digestive tract contents of herbivores that come in contact with fluoroacetate, would be important to ascertain the diversity and prevalence of microorganisms that can degrade these toxins. In the future, genes encoding enzymes from such microorganisms may be used to engineer new bacterial strains able to colonize the rumen and degrade fluoroacetate from toxic plants, thereby protecting the animal from poisoning by this compound.

This study aimed to isolate and identify bacteria able to degrade sodium fluoroacetate from soil and plant samples collected in the State of Paraíba, Brazil.

## 2. Materials and Methods

### 2.1. Samples Collection

Plant and soil samples were collected in the State of Paraíba, Brazil, in areas where *Mascagnia rigida* and *Palicourea aenofusca* were present. Soil samples were collected at the plant base, 1 to 8 cm depth. Leaves and flowers were also collected. All samples were placed in individual 50 mL Falcon type tubes and sent to the laboratory under refrigerated conditions for the immediate cultivation of associated bacteria.

### 2.2. Bacterial Isolation

Bacterial isolation was performed in 50 mL Falcon type tubes in mineral medium (Brunner) added with vitamins (http://www.dsmz.de/microorganisms/medium/pdf/DSMZ_Medium457.pdf) and 20 mmol L^−1^ sodium fluoroacetate (SF) (Sigma-Fluka) as single-carbon source. This medium will be here designated as Brunner medium. Samples were incubated at 28°C in an orbital shaker. After 48 hours, one mL of the first growth was transferred to test tubes containing nine mL of Brunner medium and incubated under the same conditions described above.

The SF defluorination was measured with an F^−^ selective electrode (Thermo Electron Corporation) in 24-well plates containing 500 *μ*L of culture and 500 *μ*L of Total Ionic Strenght Adjustment Buffer-TISAB (diaminocyclohexane, sodium chloride, and glacial acetic acid, pH 5.5). The fluoride ion released from the microbial degradation of the sodium fluoroacetate was expressed in millimoles (mmol), the defluorination rate of 20 mmol L^−1^ corresponding to the release of 20 mmol L^−1^ F^−^.

Samples showing SF defluorination were cultivated in serial dilutions from 10^−1^ to 10^−9^. To obtain pure colonies the highest dilution that presented SF defluorination was plated on Brunner agar (Brunner medium added with agar 1%) and incubated at 28°C for 72 hours. Subsequently, each colony was used to inoculate three test tubes containing 9 mL of Brunner medium, which were monitored for SF defluorination. *Pseudomonas fluorescens* (strain DSM 8341) was used as positive control for fluoroacetate dehalogenase activity. Nine mL of Brunner medium without bacterial inoculum were incubated under the same conditions to evaluate the sodium fluoroacetate degradation background.

The standard sample (strain DSM 8341) and the bacteria isolated from soil and plants were grown into Brunner medium with increased concentrations of SF (20 mmol L^−1^, 40 mmol L^−1^, 60 mmol L^−1^, and 80 mmol L^−1^ to 200 mmol L^−1^) to evaluate the highest defluorination rate. Additionally, to evaluate defluorination in the presence of other carbon sources, strain DSM 8341 and the bacteria isolated from soil and plants were also grown in Brunner medium enriched with yeast extract and glucose, on the following conditions: (1) Brunner medium alone; (2) medium supplemented with yeast extract 0.01% and glucose 2%; (3) medium with yeast extract 0.01%; (4) medium with glucose 2%.

### 2.3. 16S rRNA Gene Sequence Identification

Bacteria displaying defluorination activity were identified by polymerase chain reaction (PCR) amplification and sequencing of the 16S rRNA gene. DNA extraction was performed with Brazol (LGC Biotechnology) according to the manufacturer's specifications. 16S rRNA gene was amplified in buffer containing 0.5 *μ*M of 27f and 1492r universal primers [11], 2U of Taq DNA polymerase, 0.2 mM of dNTP and 100 ng of DNA and ultrapure water to a final volume of 20 *μ*L. In the negative control, the DNA volume was substituted by ultrapure water. The amplified products were applied into agarose gel 1% and submitted to electrophoresis. DNA was stained with ethidium bromide and bands visualized with an imaging system (UVP-Bioimaging Systems).

The sequencing reaction was performed with BigDye kit according to manufacturer's recommendations (Applied Biosystems) and the product sequenced in the Genetic Analyzer 3500 XL sequencer (Applied Biosystems).

### 2.4. Sequence Analysis and Phylogram

16S rRNA gene sequences were assembled with the CAP3 Sequence Assembly Program (http://pbil.univ-lyon1.fr/cap3.php). DNA sequences were analyzed by Basic Local Alignment Search Tool (BLAST) available on the website of the National Center for Biotechnology Information (NCBI—http://www.ncbi.nlm.nih.gov/BLAST). Species identification was based on maximum score, identity, and coverage values. The Greengenes database and workbench were used to corroborate species identification (http://greengenes.lbl.gov/). The phylogram tree was generated with MEGA 5 software using the default parameters (http://www.megasoftware.net/mega.php) [12, 13].

## 3. Results

Following the analysis of all 16S rRNA genes, seven isolates were identified as *Paenibacillus* sp. (ECPB01), *Burkholderia* sp. (ECPB02), *Cupriavidus* sp. (ECPB03), *Staphylococcus* sp. (ECPB04), *Ancylobacter* sp. (ECPB05), *Ralstonia* sp. (ECPB06), and *Stenotrophomonas* sp. (ECPB07) (Table 1). *Cupriavidus* sp. and *Ralstonia* sp. were isolated from both soil and plants samples, whereas *Paenibacillus* sp., *Burkholderia* sp. and *Ancylobacter* sp. were found from soil samples and, *Staphylococcus* sp. and *Stenotrophomonas* sp. were isolated from plant samples.

**Figure 1:**
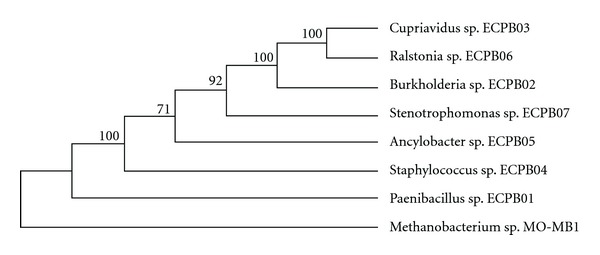
Phylogenetic tree based on 16S rRNA sequences by Maximum Parsimony analysis. ECPB01 to ECPB07 represent the isolate code and *Methanobacterium* sp. MO-MB1 (gi∣311141366∣dbj∣AB598270.1∣) the outgroup. The evolutionary history was inferred using the Maximum Parsimony method. The bootstrap consensus tree inferred from 1000 replicates is taken to represent the evolutionary history of the taxa analyzed. Branches corresponding to partitions reproduced in less than 50% bootstrap replicates are collapsed. The percentage of replicate trees in which the associated taxa clustered together in the bootstrap test (1000 replicates) is shown next to the branches. The MP tree was obtained using the Close-Neighbor-Interchange algorithm with search level 1 in which the initial trees were obtained with the random addition of sequences (10 replicates). The analysis involved 8 nucleotide sequences. All positions containing gaps and missing data were eliminated. There were a total of 1235 positions in the final dataset. Evolutionary analyses were conducted in MEGA5.

In order to further support the attribution of genera based on BLAST maximum scores, identity and coverage values, a phylogram tree was built from the sequences previously trimmed to have the same length and indicated that five isolates were phylogenetically closely related (Figure 1): *Ancylobacter* sp., three Burkholderiaceae (*Cupriavidus* sp., *Ralstonia* sp., *Burkholderia* sp.) and *Stenotrophomonas* sp. Two other species, *Paenibacillus* sp. and *Staphylococcus* sp. formed a heterogeneous group.

**Figure 2:**
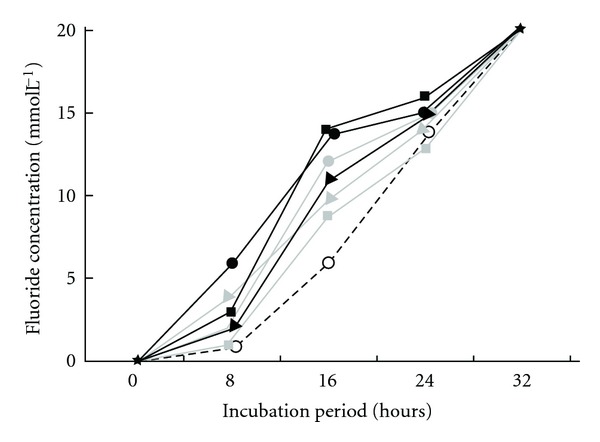
Sodium fluoroacetate degradation rate by bacteria isolated from both soil and plants in the State of Paraíba, Brazil. Symbols are as follows. The white circle: ECPB01; the black triangle: ECPB02; the black square: ECPB03; the grey circle: ECPB04; the grey square: ECPB05; the grey triangle: ECPB06; the black circle: ECPB07.

All bacterial isolates displayed SF degradation activity, reaching a 20 mmol L^−1^ level of released fluoride ion 32 hours after incubation in Brunner medium containing 20 mmol L^−1^ of SF (Figure 2). The same result was observed with the *Pseudomonas fluorescens* control strain (DSM 8341). There was no release of fluoride ions when the Brunner medium was incubated in the absence of bacteria.

When samples were grown in different concentrations of SF the maximum rate of defluorination was 140 mmol L^−1^ F^−^ in 140 mmol L^−1^ of concentration. At higher SF concentrations (160 mmol L^−1^, 180 mmol L^−1^ and 200 mmol L^−1^) the levels of degradation were still 140 mmol L^−1^. When the isolates were cultured into Brunner medium added with others carbon sources (yeast extract 0.01% and glucose 2%), an intense bacterial growth was observed after 24 hours, but SF degradation achieved only 20 mmol L^−1^ F^−^ between 48 to 64 hours with 20 mmol L^−1^ SF initial concentration.

## 4. Discussion

*Ancylobacter* sp., the Burkholderiaceae (*Cupriavidus* sp., *Ralstonia* sp. and *Burkholderia* sp.) and *Stenotrophomonas* sp. belong to the Alpha, Beta and Gamma-proteobacteria classes, respectively. The two other species, *Paenibacillus* sp. and *Staphylococcus* sp. are from *Paenibacillaceae* and *Staphylococcaceae* families, respectively, forming a heterogeneous group; the genus assignment based on BLAST values was coherent with the phylogram, which is in agreement with the phylogeny of these bacteria. The existence of a similar haloacid dehalogenase activity among distantly related microorganisms isolated over a restricted area points towards a common and effective selective pressure acting in a broad set of soil microorganisms.

Annotated genomic sequences are available at the NCBI genome database for the species *Burkholderia* sp., *Cupriavidus taiwanensis*, *Staphylococcus saprophyticus*, *Paenibacillus* sp., *Ralstonia* sp., and *Stenotrophomonas maltophilia*. The *Ancylobacter dichloromethanicus* genome has not yet been sequenced, but there is 16S rRNA sequence for this bacterial species deposited in Genbank. For the genera listed above only once was the detoxifying enzyme designated as fluoroacetate dehalogenase (Fac-Dex FA1) for *Burkholderia* sp. [14]. For *Cupriavidus* sp., *Staphylococcus* sp., *Paenibacillus* sp., *Ralstonia* sp., *Stenotrophomonas* sp., and *Ancylobacter *sp. the dehalogenases were annotated as haloacid dehalogenase hydrolase domain-containing protein [15, 16], haloacid dehalogenase-like hydrolase [17, 18], 2-haloalkanoic acid dehalogenase [17, 18], haloacetate dehalogenase [18, 19], and dichloromethane dehalogenase [20]. However, when the seven isolated were grown in the presence of SF the substrate was defluorinated. This finding may be explained by the unspecificity of the dehalogenases, which can use as substrates other compounds structurally similar to SF, which is known as cross-adaptation [21]. Liu and colleagues [22] verified that fluoroacetate dehalogenase degrades other halogenated compounds such as chloroacetate, bromoacetate, iodoacetate, and dichloroacetate. Similar results were obtained by Donnelly and Murphy [23] that found fluoroacetate dehalogenase activity catalyzing chloroacetate, bromoacetate, and ethyl fluoroacetate. Also sodium fluoroacetate can be defluorinated by L-2 haloacid dehalogenase [24]. Another possibility is the lateral transfer of fluoroacetate dehalogenase genes.

Although a couple of other environmental bacteria such as *Moraxella* sp. [25], *Acinetobacter*, *Arthrobacter*, *Aureobacterium*, *Bacillus*, *Pseudomonas*, *Weeksella*, and *Streptomyces* [8] also present fluoroacetate dehalogenase activity, there are no previous reports on such an activity from microorganisms belonging to any of the six species described here: *Cupriavidus* sp., *Staphylococcus* sp., *Paenibacillus* sp., *Ralstonia* sp., *Stenotrophomonas* sp., and *Ancylobacter* sp., since genes for haloacid dehalogenases were annotated in the genomes of the first five species. It is therefore reasonable to assume that all seven isolates have the fluoroacetate genes in their chromosomes and not in plasmids, which further support the existence of a strong, durable and common environmental selective pressure over these organisms.

In all isolates defluorination occurred up to 32 hours of cultivation, with the release of 20 mmol L^−1^ fluoride ion in the presence of 20 mmol L^−1^ sodium fluoroacetate. These results were similar to those reported by Davis and colleagues [26] with *Burkholderia *sp., which also degraded SF in 32 hours, releasing 20 mmol L^−1^ F^−^ with 20 mmol L^−1^ of SF.

Growing the strains in different concentrations of SF indicated that the highest degradation rate was 140 mmol L^−1^ F^−^ with 140 mmol L^−1^ of substrate. For higher fluoride concentrations the defluorination rate did not increase, which may be due to exhaustion of other nutrients.

When the bacteria were cultivated in Brunner medium added with yeast extract and glucose, the dehalogenase activity was delayed, probably due to the availability of the others energy sources and to the difficult cleavage of the strong carbon-fluorine bond in fluoroacetate [7]. In conclusion, seven fluoroacetate degrading bacteria were isolated from both soil and plants, six of which were not previously reported as able to degrade SF. The possibility to use some of these bacteria to establish in the rumen an engineered bacterial population able to degrade fluoroacetate and protect ruminants from the poisoning by this compound should be explored in the future.

## Figures and Tables

**Table 1:** Results of BLAST for the 16S rRNA sequences obtained from bacteria isolated from soil and plants samples.

Isolate code no.	Most similar species*	Isolated from	16S rRNA sequence length	Coverage (%)	Max score	*E*-value	Identity (%)
ECPB01	*Paenibacillus* sp.	Soil	1425	99	2619	0.0	99
ECPB02	*Burkholderia* sp.	Soil	1398	99	2553	0.0	99
ECPB03	*Cupriavidus *sp.	Soil and plant	1398	99	2540	0.0	99
ECPB04	*Staphylococcus* sp.	Plant	1402	100	2569	0.0	99
ECPB05	*Ancylobacter* sp.	Soil	1368	99	2435	0.0	99
ECPB06	*Ralstonia* sp.	Soil and plant	1407	99	2551	0.0	99
ECPB07	*Stenotrophomonas* sp.	Plant	1417	99	2606	0.0	99

*Genera were identified based on maximum score, identity, and coverage.
